# BCRP expression in schwannoma, plexiform neurofibroma and MPNST

**DOI:** 10.18632/oncotarget.21075

**Published:** 2017-09-16

**Authors:** Maurits de Vries, Olaf van Tellingen, Andel. G.L. van der Mey, Antonius M.G. Bunt, Inge Briaire-de Bruijn, Pancras C.W. Hogendoorn

**Affiliations:** ^1^ Department of Pathology, Leiden University Medical Center, Leiden, The Netherlands; ^2^ Department of Otolaryngology, Leiden University Medical Center, Leiden, The Netherlands; ^3^ Division of Pharmacology/Mouse Cancer Center, The Netherlands Cancer Institute, Amsterdam, The Netherlands; ^4^ IzumiBiosciences, Inc., Lexington, USA

**Keywords:** BCRP, schwannoma, neurofibroma, drug resistance, MPNST

## Abstract

**Background:**

peripheral nerve sheath tumors comprise a broad spectrum of neoplasms. Vestibular schwannomas and plexiform neurofibromas are symptomatic albeit benign, but a subset of the latter pre-malignant lesions will transform to malignant peripheral nerve sheath tumors (MPNST). Surgery and radiotherapy are the primary strategies to treat these tumors. Intrinsic resistance to drug therapy characterizes all three tumor subtypes. The breast cancer resistance protein BCRP is a transmembrane efflux transporter considered to play a key role in various biological barriers such as the blood brain barrier. At the same time it is associated with drug resistance in various tumors. Its potential role in drug resistant tumors of the peripheral nervous system is largely unknown.

**Objective:**

to assess if BCRP is expressed in vestibular schwannomas, plexiform neurofibromas and MPNST.

**Material and methods:**

immunohistochemical staining for BCRP was performed on a tissue microarray composed out of 22 vestibular schwannomas, 10 plexiform neurofibromas and 18 MPNSTs.

**Results:**

sixteen out of twenty-two vestibular schwannomas (73%), nine out of ten plexiform neurofibromas (90%) and six out of eighteen MPNST (33%) expressed BCRP in the vasculature. Tumor cells were negative.

**Conclusion:**

BCRP is present in the vasculature of vestibular schwannomas, plexiform neurofibromas and MPSNT. Therefore, it may reduce the drug exposure of underlying tumor tissues and potentially cause failure of drug therapy.

## INTRODUCTION

Peripheral nerve sheath tumors (PNST) are relatively common neoplasm’s that comprise a broad spectrum of different subtypes. Most of these tumors are histologically benign such as schwannomas and neurofibromas [1, 2]. Next to these benign tumors there is a subset of malignant lesions like the malignant peripheral nerve sheath tumors (MPNST) [3, 4]. Neurofibromas, MPNST and schwannomas are examples of PNST that occur either sporadically or as part of hereditary neurocutaneous diseases like neurofibromatosis type I (NF1) and neurofibromatosis type II (NF2) respectively. Both these disorders seem to result from the inactivation of a classic tumor suppressor gene. Neurofibromas and MPNST show loss of *NF1* expression. The *NF1* gene is located on chromosome 17q11.2. and encodes the tumor suppressor protein neurofibromin [5]. NF1 is caused by germline mutations in *NF1* but there are also mosaic forms of this disease [6]. MPNST or plexiform neurofibromas without other symptoms of NF1, i.e. sproradic tumors, are probably caused by somatic mosaicism for an *NF1* mutation. A similar situation is seen in NF2. NF2 is caused by biallilec inactivation of the *NF2* gene, located on chromosome 22q11, which encodes the tumor suppressor protein merlin [7]. Bilateral vestibular schwannomas are pathognomonic for this rare disease. However, most vestibular schwannomas occur as sporadic unilateral tumors [8].

Schwannomas occur in a wide range of anatomical sites, including the subcutaneous tissues of the distal extremities and the head and neck region. Schwannomas in the head and neck region have a predilection to derive from the vestibular portion of the eighth cranial nerve, better known as vestibular schwannomas (VS). Schwannomas are neoplastic proliferations that exclusively comprise Shwann cells while neurofibromas contain multiple cell types such as perineurial cells, fibroblasts and to a lesser extent Schwann cells [1, 9–11] There are two types of neurofibromas: dermal and plexiform. Plexiform neurofibromas are strongly related to NF1, affecting 20% to 40% of patients suffering from this condition [12, 13]. These tumors often occur in the head, skull base, or neck but also manifest themselves on the trunk and limbs. Plexiform neurofibromas have the potential to transform into MPNST. However, not all MPNST develop from pre-existing neurofibromas, as approximately half of all MPNST arise sporadically without a known precursor [14].

These different types of tumors require different types of therapy. To date surgical excision is the only effective treatment for plexiform neurofibromas, but recent literature demonstrated that targets for pharmacological treatment are emerging [15, 16]. Therapeutic management of (vestibular) schwannomas consist of surgery or radiotherapy and pharmacological treatment options were recently tested as well [17]. Although there are promising results in individual NF2 patients treated with targeted therapy there are also reports indicating drug resistance in these tumors [18]. A similar situation exists in MPNST. The triad of surgery, radiotherapy and multi-agent chemotherapy is applied to these tumors as well. Despite promising reports on for instance (neoadjuvant) doxorubicin-ifosfamide treatment regimens, these tumors are often characterized by a highly aggressive behavior and resistance to multidrug therapy, resulting in poor long-term survival rates [19–21]. In short, despite the different therapies that are applied to these PNST one of their common dominators is the fact that they show a certain degree of drug resistance.

**Figure 1:**
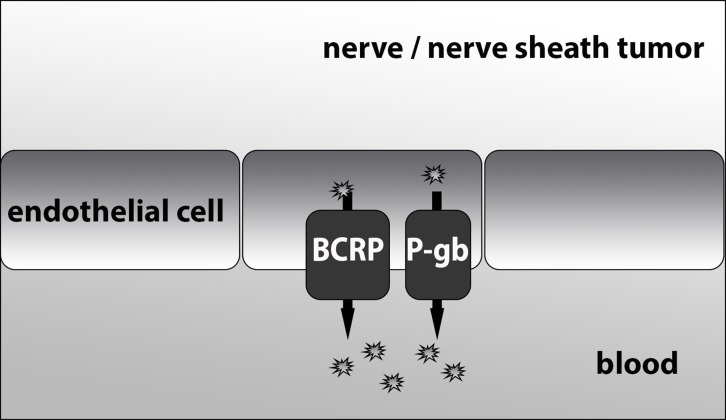
A mechanistic figure of the proposed function of BCRP and P-gb at the blood-nerve-barrier

Acquired and/or innate drug resistance of tumor cells is a common phenomenon and a major hurdle to effective chemotherapeutic intervention. An important mechanism contributing to drug resistance concerns the expression of ATP binding cassette (ABC) transporter proteins that are capable of extruding drugs from tumors [22]. These energy-dependent transmembrane proteins transport a wide range of substrates, including many anticancer drugs, across cell membranes [23–27]. So far 49 genes have been identified to encode for members of the ABC transporter family [28], but only a subset of these is involved in drug resistance. Of these drug transporters ABCB1 (P-gp) and ABCG2 (BCRP) are the most extensively studied. They were first discovered in tumor cells [29, 30], but are also expressed at the apical membranes of epithelial cells in biological barrier tissues such as in the intestines, kidneys and liver and have an important role in the clearance of xenobiotics from the body [23]. In addition, they are expressed in specialized endothelial cells that form the blood-brain, blood-testis and blood-placenta barriers where they help to limit the exposure of the underlying tissues (brain, testis and fetus) to xenobiotics [31–33]. Besides efflux transporters, these specialized endothelial cells also present other barrier properties, such as tight junctions and lack of fenestrations that limit para-cellular entry of drugs. In the brain, the surrounding glial cells (astrocytes, pericytes) govern the expression of these barrier markers in these endothelial cells. The blood-brain barrier (BBB) may thereby “protect” tumor cells that reside within the central nervous system [34]. Similar to the situation in the brain, tumors originating from the peripheral nerve sheath may be protected by the so called blood-nerve-barrier (BNB). Our hypothesis is that the blood-nerve-barrier might hinder drugs from reaching their target cells in peripheral nerve sheath tumors, thereby contributing to drug resistance (Figure 1). The three tumor types we included in this analysis were selected because all of them originate from the peripheral nerve sheath and, as mentioned earlier, each of them are characterized by some form of drug resistance. The BNB is located in microvasculature of the endoneurium and the inner most layers of the perineurium [35] and there are reports that, analogous to the situation at the BBB, the BNB contains members of the ABC transporter family such as BCRP and P-gb [36, 37]. Apart from the concept of protection by the BNB, these tumors may also be drug resistant because the tumor cells themselves express ABC drug transporters. Since its discovery, BCRP expression has been observed in several types of tumors [38–47] and elevated expression levels of this transporter have been correlated with poor prognosis in a number of studies [48–50]. Moreover, the expression of BCRP in tumor cells has been associated with a rare subset of so-called cancer stem cells, similar to the expression of BCRP in normal stem cells [51, 52]. Consequently, the expression of BCRP both in tumor blood vessels or in tumor cells can mediate drug resistance.

The aim of this study was to assess the presence and localization of BCRP in peripheral nerve sheath tumors. We investigated the expression pattern of BCRP in twenty-two sporadic vestibular schwannomas, ten plexiform neurofibromas and eighteen MPNST using an immunohistochemical assay performed on a tissue microarray (TMA) composed of these tumors. We used TMA technology because it provides the advantage of simultaneously analyzing a large panel of tumors with a high degree of experimental standardization [53]. Is has also been shown that the clinico-pathological findings obtained by this technique are highly representative of their donor tissues [54]. The results of our analysis show that BCRP is expressed in all three of these tumor types. This observation indicates that BCRP might reduce drug accumulation in these peripheral nerve sheath tumors thus creating a hurdle to effective drug treatment.

## RESULTS

**Figure 2:**
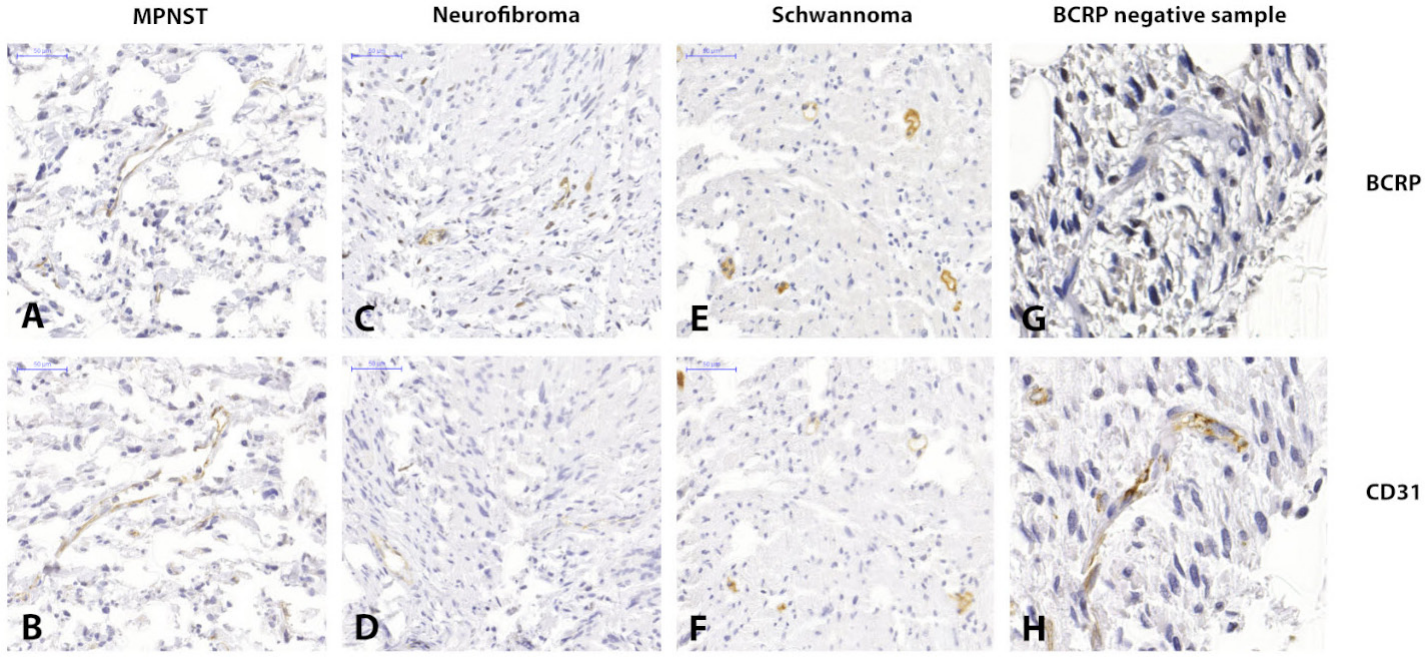
Immunohistochemistry images showing clear BCRP and CD31 positivity in the microvascular epithelium of MPNST**(A** and **B),** plexiform neurofibroma **(C** and **D)** and vestibular schwannoma **(E** and **F)** respectively. Images (**G** and **H**) show a CD31 positive yet BCRP negative sample of a vestibular schwannoma.

The results from this immunohistochemical, tissue micro array based study indicate the presence of BCRP in the microvascular endothelium of MPNST, plexiform neurofibromas and vestibular schwannomas (Figure 2). Six out of the eighteen MPNST samples showed vascular BCRP expression. Two of the positive specimens were NF1 related and the other four were sporadic tumors. Tumor cells were negative for BCRP. One of the studied MPNSTs was a recurrent tumor and matched with another specimen in this study. In both cases, the vasculature of these samples was BCRP negative. None of the MPNST patients received chemotherapy prior to resection. The vasculature of nine out of ten plexiform neurofibroma samples was BCRP positive as well as the vasculature of sixteen of the twenty-two schwannomas. Two separate plexiform neurofibroma samples originated from the same NF1 patient and both these tumors had BCRP positive vasculature. Unfortunately, the analyzability of some of the MPNST tumor specimens on the TMA slide was impaired due to necrosis. We found that in four out of eighteen MPNST cases one or two specimens showed intrinsic tumor necrosis making them unsuitable for microscopic analysis. Nevertheless, at least one of the three specimens of these tumors contained representative tumor tissue. Therefore it was still possible to perform adequate microscopic analysis on tissue from all the tumors in the analysis. Of these four MPNST one was scored positive for BCRP and the other three were scored negative.

## DISCUSSION

Present treatment of peripheral nerve sheath tumors is mainly a surgical matter. The unraveling of the underlying molecular pathologies and the ongoing development of new therapeutic agents may provide potentially effective drugs as an alternative- or concomitant therapeutic strategy. Unfortunately, however, the occurrence of innate or acquired drug resistance of tumors is a common event. Drug resistance is a frequently encountered problem in MPNST, but it is also observed in benign or precursor lesions. Multidrug resistance is a complex phenomenon and frequently multifactorial. One important reason is impaired drug delivery to the target tissues because of the expression of drug efflux proteins in the (micro) vasculature of tumor tissues or because of expression of these proteins by tumor cells themselves. In central nervous system (CNS) tissues, the restricted entry is due to drug transporters located at the interface between the blood and the brain (the BBB). A similar situation may be present at the interface between blood and peripheral nerves, but this has not been well established yet. The most extensively studied efflux transporters of the BBB are P-gp (ABCB1) [55] and BCRP (ABCG2) [56]. Together, these two efflux transporters team up to restrict the CNS penetration of a wide range of substrates including many potentially useful drugs [56–59]. In this study, we have investigated the expression of BCRP in tumors of the peripheral nervous system. Based on our results it is not entirely clear if the vascular BCRP expression we observed in a selection of the investigated tumors is a specific characteristic of these tumors, or if it is a remaining part of the blood-nerve-barrier. Dahin et al [60] identified BCRP expression in retinal nerve fibers suggesting that BCRP is part of the blood-nerve-barrier that protects retinal nerve fibers from injury by removing intracellular toxins and xenobiotics. A contradictory observation was made by Huang et al [61]. They investigated BCRP and P-gp in peripheral nerves using a tissue distribution assay on rats but did not find a difference in drug distribution between wild type- or BCRP/P-gp knock out rats. However, in the Huang study the Abcg2 KO had little effect on the brain distribution of known BCRP substrate drugs and these findings are at odds with other ABC KO studies and have not been replicated independently since [37, 62, 63]. Furthermore, they are in stark contrast with multi drug resistance observed in clinical studies in NF1 patients with known P-gp and/or BCRP substrate drugs [15, 16, 64–66]. Similarly, prior studies in MPNST confirmed the presence of drug resistant sarcoma stem cells [67, 68] and the P-gp and BCRP efflux pumps [47, 50, 69, 70], while drug trials in MPNST patients have not improved outcomes [50, 71] Our findings provide a rationale to further study the hypothesis that endothelial BCRP expression may be part of the reason why drug therapy of PNST often fails [47]. If this hypothesis is correct it could mean that inhibition of BCRP may aid in rendering these tumors more susceptible to drug therapy. A potential strategy to achieve this is to co-administer elacridar, a potent, selective inhibitor of both P-gp and BCRP with molecularly targeted drugs to enhance drug levels in diseased neural tissues and improve outcomes as has been observed in animal models of other pump-protected diseases [34, 72–82].

In conclusion, our results demonstrate the expression of BCRP in the vascular endothelium in a substantial fraction of MPNST, plexiform neurofibromas and sporadic vestibular schwannomas. Similar to CNS tumors, the presence of BCRP, and perhaps other members of the ABC efflux transporter family, may reduce the drug exposure of underlying tumor tissues and mediate resistance to drug therapy.

## MATERIALS AND METHODS

### Patients

The cases included in this study were retrospectively selected from the files of the bone- and soft tissue tumor database at the department of Pathology of the Leiden University Medical Center, Leiden the Netherlands. Tumor specimens were obtained from patients surgically treated for their tumors between January 1999 and December 2012. Formalin-fixed paraffin-embedded samples of twenty-two sporadic vestibular schwannomas, ten plexiform neurofibromas and eighteen MPNST were selected. Of these selected tumors two separate plexiform neurofibromas originated from the same patient and one MPNST was a recurrence of a primary tumor included in this analysis as well. Surgery was performed at the departments of Neurosurgery, Otolaryngology, Orthopedic surgery and General surgery of the Leiden University Medical Center. In each case the diagnosis was made according to the WHO classification of soft tissue tumors [83]. All tumor samples were handled in a coded fashion and all procedures were performed according to the ethical guidelines of the Code for Proper Secondary Use of Human Tissue in The Netherlands (Dutch Federation of Medical Scientific Societies). Additional clinicopathological data are shown in Supplementary Table 1.

### Tissue microarray (TMA) preparation

Preparation of the TMAs was performed at the department of pathology of the Leiden University Medical Center. TMAs were constructed from 1mm cores of all tumor samples using a TMA Master (3DHISTECH Ltd, Budapest, Hungary). Per tumor three randomly selected cores were included in the TMA in order to compensate for intra-tumoral heterogeneity. Normal colon, tonsil, placenta, prostate and spleen tissue together with mamma carcinoma were used to serve as internal controls and points of orientation. In line with data provided by the manufacturer we found high BCRP expression in placenta and low expression in colon tissue.

### Immunohistochemistry (IHC)

Immunohistochemical reactions were performed according to standard laboratory methods [84]. In brief, heat-induced antigen retrieval was performed after dewaxing and rehydration, followed by blocking of endogenous peroxidase with 3% H_2_O_2_ in methanol. Incubation with the primary antibodies BCRP (Abcam; ab24115) and CD31 (Abcam; ab28364) was overnight. Subsequently, CD31 sections were conjugated with Labelled Polymer-HRPAnti-Rabbit Envision (DakoCytomation; K4005) while conjugation of the BCRP sections was performed with Goat-α-Rat-Bio (Santa Cruz; SC-2041) and Streptavidin/HRP (DakoCytomation; P0397) respectively. Visualization was carried out with a diaminobenzidine solution. All washing procedures were conducted in phosphate-buffered saline. Slides were counterstained with haematoxylin.

### Microscopic analysis

After staining the TMA was scanned using a Pannoramic MIDI Digital Slide Scanner (3DHISTECH Ltd, Budapest, Hungary). Analysis of the digital slides took place with Pannoramic Viewer software version 1.15.3. Scoring was performed by two observers who were unaware of the clinico-pathological data. Staining of tumor specimens was classified as either positive or negative. Differently assessed cases were discussed to reach consistent scoring results.

## SUPPLEMENTARY MATERIALS TABLE





## Abbreviations

ABC: ATP binding cassette
ABCB1: ATP-binding cassette sub-family B member 1
ABCG2: ATP-binding cassette sub-family G member 2
BBB: Blood-brain-barrier
BCRP: Breast Cancer Resistance Protein
BNB: Blood-nerve-barrier
MPNST: Malignant peripheral nerve sheath tumors
NF 1: Neurofibromatosis type I
NF 2: Neurofibromatosis type II
P-gb: permeability glycoprotein
PNST: Peripheral nerve sheath tumors
TMA: Tissue micro array
VS: Vestibular schwannomas
