# Supplementary material for: BCRP expression in schwannoma, plexiform neurofibroma and MPNST
Source: Oncotarget. 2017 Sep 16;8(51):88751–9. doi: 10.18632/oncotarget.21075 (PMC5687642; doi:10.18632/oncotarget.21075)
Supplement: Supplementary file 1 [file oncotarget-08-88751-s001.pdf]

BCRP expression in schwannoma, plexiform neurofibroma and MPNST

SUPPLEMENTARY MATERIALS

Supplementary Table 1: Clinicopathological data

See Supplementary File 1
