# Supplementary material for: BCRP expression in schwannoma, plexiform neurofibroma and MPNST
Source: Oncotarget. 2017 Sep 16;8(51):88751–9. doi: 10.18632/oncotarget.21075 (PMC5687642; doi:10.18632/oncotarget.21075)
Supplement: Supplementary file 2 [file oncotarget-08-88751-s002.docx]

| specimen | tumor type | sex | age (yrs.) | tumor localisation | | BRCP |  |
| --- | --- | --- | --- | --- | --- | --- | --- |
| L1399 | NF1 related MPNST | F | 16 | upper leg, right | positive | | |
| L4304 | NF1 related MPNST | F | 29 | second thoracic vertebra, left | negative | | |
| L4309 | NF1 related MPNST | F | 15 | mandible angle, right | positive | | |
| L4326 | NF1 related MPNST | F | 27 | flank region, left | negative | | |
| L1537 | sporadic MPNST* | M | 22 | back, middle | negative | | |
| L1448 | sporadic MPNST | M | 51 | inguinal region, left | negative | | |
| L1219 | sporadic MPNST | F | 35 | gluteus region, right | negative | | |
| L1503 | sporadic MPNST | F | 58 | upper leg, right | negative | | |
| L1509 | sporadic MPNST | M | 17 | upper arm, left | negative | | |
| L1867 | sporadic MPNST | M | 57 | upper leg, left | negative | | |
| L2056 | sporadic MPNST | M | 24 | inguinal region, left | positive | | |
| L2170 | sporadic MPNST | M | 22 | brachial plexus, left | negative | | |
| L4303 | sporadic MPNST | F | 41 | brachial plexus, left | positive | | |
| L4320 | sporadic MPNST | M | 22 | back, middle | negative | | |
| L4322 | sporadic MPNST | F | 48 | brachial plexus, left | negative | | |
| L4325 | sporadic MPNST | F | 35 | fifth cervical vertebra, left | negative | | |
| L4327 | sporadic MPNST | M | 68 | retroauricular region, right | positive | | |
| L4328 | sporadic MPNST | V | 26 | fifth cervical vertebra, left | positive | | |
| L4305 | NF1 related plexiform neurofibroma | F | 33 | foot, right | negative | | |
| L4321 | NF1 related plexiform neurofibroma | M | 23 | skin of neck, left | positive | | |
| L4330 | NF1 related plexiform neurofibroma | V | 42 | supraclavicular region, right | positive | | |
| L4331 | NF1 related plexiform neurofibroma | M | 30 | upper leg, right | positive | | |
| L4332 | NF1 related plexiform neurofibroma** | M | 30 | occipital region, middle | positive | | |
| L4333 | NF1 related plexiform neurofibroma | M | 24 | cheek, left | positive | | |
| L4335 | NF1 related plexiform neurofibroma | F | 31 | neck region, left | positive | | |
| L4302 | sporadic plexiform neurofibroma | F | 27 | median nerve left | positive | | |
| L4329 | sporadic plexiform neurofibroma | V | 26 | axilla, right | positive | | |
| L4334 | sporadic plexiform neurofibroma | F | 51 | femoral nerve, right | positive | | |
| L1493 | sporadic schwannoma | F | 39 | cerebellopontine angle, right | positive | | |
| L3580 | sporadic schwannoma | M | 58 | cerebellopontine angle, right | negative | | |
| L3583 | sporadic schwannoma | M | 47 | cerebellopontine angle, left | positive | | |
| L3586 | sporadic schwannoma | F | 48 | cerebellopontine angle, right | negative | | |
| L3590 | sporadic schwannoma | M | 43 | cerebellopontine angle, right | positive | | |
| L3593 | sporadic schwannoma | F | 53 | cerebellopontine angle, left | positive | | |
| L3604 | sporadic schwannoma | F | 43 | cerebellopontine angle, right | positive | | |
| L4306 | sporadic schwannoma | M | 69 | cerebellopontine angle, left | positive | | |
| L4307 | sporadic schwannoma | F | 57 | cerebellopontine angle, right | positive | | |
| L4308 | sporadic schwannoma | M | 73 | cerebellopontine angle, right | positive | | |
| L4310 | sporadic schwannoma | F | 59 | cerebellopontine angle, right | positive | | |
| L4311 | sporadic schwannoma | F | 56 | cerebellopontine angle, left | positive | | |
| L4312 | sporadic schwannoma | F | 56 | cerebellopontine angle, left | positive | | |
| L4313 | sporadic schwannoma | M | 43 | cerebellopontine angle, right | negative | | |
| L4314 | sporadic schwannoma | F | 59 | cerebellopontine angle, left | positive | | |
| L4315 | sporadic schwannoma | F | 61 | cerebellopontine angle, right | positive | | |
| L4316 | sporadic schwannoma | F | 67 | cerebellopontine angle, left | positive | | |
| L4317 | sporadic schwannoma | F | 50 | cerebellopontine angle, left | positive | | |
| L4318 | sporadic schwannoma | M | 55 | cerebellopontine angle, left | negative | | |
| L4319 | sporadic schwannoma | F | 72 | cerebellopontine angle, right | positive | | |
| L4323 | sporadic schwannoma | M | 62 | cerebellopontine angle, right | negative | | |
| L4324 | sporadic schwannoma | F | 54 | cerebellopontine angle left | negative | | |
| * recurrent tumor from specimen L4325; ** separate tumor from the same patient as L4331 | | | | |  | | |
